# Efflux Pump-Driven Antibiotic and Biocide Cross-Resistance in *Pseudomonas aeruginosa* Isolated from Different Ecological Niches: A Case Study in the Development of Multidrug Resistance in Environmental Hotspots

**DOI:** 10.3390/microorganisms8111647

**Published:** 2020-10-24

**Authors:** Anteneh Amsalu, Sylvia A. Sapula, Miguel De Barros Lopes, Bradley J. Hart, Anh H. Nguyen, Barbara Drigo, John Turnidge, Lex EX Leong, Henrietta Venter

**Affiliations:** 1 UniSA Clinical and Health Sciences, Health and Biomedical Innovation, University of South Australia, Adelaide, SA 5000, Australia; anteneh_amsalu.geremew@mymail.unisa.edu.au (A.A.); sylvia.sapula@unisa.edu.au (S.A.S.); miguel.debarroslopes@unisa.edu.au (M.D.B.L.); Brad.Hart@unisa.edu.au (B.J.H.); Hong.Nguyen@unisa.edu.au (A.H.N.); Lex.Leong@sa.gov.au (L.E.L.); 2 Department of Medical Microbiology, University of Gondar, Gondar 196, Ethiopia; 3 Future Industries Institute, University of South Australia, Mawson Lakes, SA 5095, Australia; Barbara.Drigo@unisa.edu.au; 4 EUCAST Development Laboratory, Adelaide Medical School, University of Adelaide, Adelaide, SA 5000, Australia; jturnidge@gmail.com; 5 Microbiology and Infectious Diseases, SA Pathology, Adelaide, SA 5000, Australia

**Keywords:** antimicrobial resistance, biocide resistance, efflux pump, *Pseudomonas aeruginosa*, cross-resistance, resistance development, selective pressure

## Abstract

*Pseudomonas aeruginosa* is an opportunistic pathogen displaying high intrinsic antimicrobial resistance and the ability to thrive in different ecological environments. In this study, the ability of *P. aeruginosa* to develop simultaneous resistance to multiple antibiotics and disinfectants in different natural niches were investigated using strains collected from clinical samples, veterinary samples, and wastewater. The correlation between biocide and antimicrobial resistance was determined by employing principal component analysis. Molecular mechanisms linking biocide and antimicrobial resistance were interrogated by determining gene expression using RT-qPCR and identifying a potential genetic determinant for co- and cross-resistance using whole-genome sequencing. A subpopulation of *P. aeruginosa* isolates belonging to three sequence types was resistant against the common preservative benzalkonium chloride and showed cross-resistance to fluoroquinolones, cephalosporins, aminoglycosides, and multidrug resistance. Of these, the epidemiological high-risk ST235 clone was the most abundant. The overexpression of the MexAB-OprM drug efflux pump resulting from amino acid mutations in regulators MexR, NalC, or NalD was the major contributing factor for cross-resistance that could be reversed by an efflux pump inhibitor. This is the first comparison of antibiotic-biocide cross-resistance in samples isolated from different ecological niches and serves as a confirmation of laboratory-based studies on biocide adapted isolates. The isolates from wastewater had a higher incidence of multidrug resistance and biocide-antibiotic cross-resistance than those from clinical and veterinary settings.

## 1. Introduction 

*Pseudomonas aeruginosa* is a ubiquitous bacterium of environmental origin that is responsible for difficult-to-treat nosocomial infections such as wound, urinary tract, and other infections [1,2,3,4]. It is also a major cause of respiratory infections and the main cause of mortality in cystic fibrosis patients [5]. *P. aeruginosa* is a highly adaptive and robust organism that can thrive in a wide range of environmental niches owing to its large and dynamic genome that provides extraordinary metabolic versatility and genetic plasticity [6]. *P. aeruginosa* strains display high intrinsic antimicrobial resistance as well as the ability to tolerate a variety of biocides such as antiseptics, disinfectants, and preservatives [6,7,8]. Recently, *P. aeruginosa* has been classified by the World Health Organization as an organism of the most critical priority in need of new drug development. This is due to the global emergence of multidrug-resistant (MDR) high-risk clones, which are resistant to almost all available antipseudomonal drugs in clinical settings [9,10].

Among the antipseudomonal drugs, fluoroquinolones (FQs) are among the first line of antimicrobials used to treat *P. aeruginosa* infections [11,12]. FQs are potent, broad-spectrum antimicrobial agents with excellent bioavailability [8] and are characterized by a high degree of persistence in the environment [13]. Several studies have investigated the association between increased consumption of FQs and FQ resistance among *P. aeruginosa* isolates [14,15], while in vitro experiments have also demonstrated biocide exposure as a driver of antimicrobial resistance [16,17]. Biocides, which include disinfectants, antiseptics, and preservatives, are widely used as a part of infection prevention and control for various applications in both medical and household products [18]. For example, benzalkonium chloride (BKC) is widely used for the disinfection of surfaces and instruments [19] and also as a preservative in non-sterile formulations [20]. Triclosan is used in hand soaps and a large variety of everyday products such as toothpaste, mouthwash, deodorant, shower gel, hand lotion, hand cream, and hand sanitizer [21]. Chlorhexidine is widely used in healthcare for preoperative decontamination and in oral health antiseptics [7]. The use of such biocides unavoidably results in the generation and release of long-lasting residues [19]. The exposure of bacteria to such biocides at subinhibitory concentrations altered the microbial community composition [16,17,22,23] and increased tolerance to biocides as well as resistance to the most critically important antimicrobials used for the treatment of resistant Gram-negative bacteria such as FQs and beta-lactams [16,17,24,25,26]. Such co-selection may result from a number of antimicrobial-resistant (AMR) mechanisms, including drug extrusion by multidrug efflux pumps and mutation of the drug target encoding genes [16,17].

In *P. aeruginosa*, intrinsic resistance is largely attributed to the expression of multidrug efflux pumps belonging to the Resistance-Nodulation-Division (RND) family [27,28,29]. These pumps are chromosomally encoded membrane proteins forming tripartite complexes composed of an inner membrane transporter protein, a periplasmic adapter protein, and an outer-membrane channel protein [30,31]. Together, these proteins form a highly effective efflux pump able to expel a wide spectrum of structurally unrelated antimicrobial agents from the cell [28]. Genomic analysis has identified structural genes for at least 12 RND type efflux systems, of which four pumps MexAB-OprM, MexCD-OprJ, MexEF-OprN and MexXY-OprM have been confirmed to play a role in MDR [32]. Of these, the MexAB-OprM is the most promiscuous and constitutively expressed multidrug efflux pump in *P. aeruginosa* and confers basal resistance to a wide range of antimicrobials [28,33]. Exposure to certain substrates or stresses leads to overexpression of the MexAB-OprM efflux pump (predominantly through mutations in regulatory genes such as *mexR, nalC*, or *nalD*) [34]. As a result of the promiscuous substrate profile of this efflux pump, the reduced accumulation of many different compounds including antibiotics, dyes, detergents, and disinfectants will be observed, leading to MDR *P. aeruginosa* [33,35]. 

In combination with the overexpression of multidrug efflux pumps, repertoires of chromosomal mutations in the quinolone-resistance-determining regions of DNA gyrase and topoisomerase IV-encoding genes *gyrA*, *gyrB*, *parC,* and *parE* contribute for high-level FQ resistance among *P. aeruginosa* isolates [36,37,38]. An in vitro experimental study demonstrated that mutations in the *gyrA* gene combined with overexpression of the MexAB-OprM efflux pump are associated with a high-level resistance to FQ [>16 times increase in FQ minimum inhibitory concentration (MIC) compared to wild type] [24,38].

The highly adaptive and persistent nature of *P. aeruginosa* [6] coupled with the frequent use of biocides and antimicrobials in healthcare settings exert a further selective pressure on *P. aeruginosa* to acquire antimicrobial resistance. In particular, wastewater from healthcare sites has been defined as a “hot spot” for the acquisition of antimicrobial resistant determinants [39], as it serves as a reservoir for both antimicrobial residues and bacteria, presenting the perfect environment for the co-selection and development of antimicrobial resistance [19,24,40,41]. Although *P. aeruginosa* is known to be intrinsically resistant to biocides such as triclosan [42], epidemiologic cut-off values (ECOFF) for biocides are not currently available through the European Committee on Antimicrobial Susceptibility Testing (EUCAST) or Clinical and Laboratory Standards Institute (CLSI), making the determination of biocide resistance difficult.

While the link between increased biocide tolerance and antimicrobial cross-resistance has been experimentally documented in *P. aeruginosa* [17,24,43], these laboratory-based findings have not yet been observed in diverse natural niches (clinical, veterinary, and wastewater). This provides evidence of a relationship between biocide use and cross-resistance to clinically used antibiotics.

The aim of this study was to assess the correlation between biocide and antimicrobial resistance on a diverse sample set of *P. aeruginosa* isolates collected from clinical, veterinary, and wastewater sources. Biochemical and in-depth bioinformatic analysis of whole genome sequencing data was employed to investigate the molecular mechanism linking increased biocide resistance to antimicrobial resistance.

## 2. Materials and Methods

### 2.1. Source and Identification of P. aeruginosa Isolates

A total of 147 *P. aeruginosa* isolates were collected: 89 from humans, 20 from companion animals, and 38 from wastewater sources. Clinical isolates were collected from 2012 to 2017 at two hospitals in South Australia, from the USA and from the Netherlands from various clinical infections [44]. Animal isolates were collected in 2013 (Australian collection) from canine otitis, and wastewater isolates were collected from healthcare-generated wastewater in South Australia. Each isolate was sub-cultured on Columbia agar plates and identified by matrix-assisted laser desorption/ionization time-of-flight mass spectrometry (MALDI-TOF) (Bruker Daltonik GmbH, Bremen, Germany). All confirmed isolates were stored at −80 °C for further microbiological and molecular studies.

### 2.2. Antimicrobial Susceptibility Testing

The minimum inhibitory concentrations (MICs) for antimicrobials were determined by the broth microdilution technique as described by the EUCAST guidelines [45] and adapted for biocides. Antimicrobial susceptibility was determined according to the 2020 EUCAST (http://www.eucast.org/) ECOFF value. The following classes of antimicrobials and biocides were used: Cephalosporins: ceftazidime, cefepime; Aminoglycosides: gentamicin, tobramycin; Fluoroquinolones: ciprofloxacin, levofloxacin; Carbapenems: imipenem, meropenem; Polymyxins: colistin; Biocides: chlorhexidine digluconate, triclosan, and benzalkonium chloride. Isolates displaying resistance to three or more antimicrobial classes were considered to be MDR [46]. *Escherichia coli* ATCC 25922 and *P. aeruginosa* ATCC 27853 were included in each experiment as control strains.

#### Establishing Biocide Epidemiologic Cut-off Values

The MICs of biocides were determined in triplicate using the broth microdilution technique. ECOFF (with 95% cut-off) values were calculated using the ECOFF finder XL 2010 program (https://clsi.org/meetings/microbiology/ecoffinder/).

### 2.3. Efflux Pump Inhibition Using a Checkerboard Assay

MICs of biocides and antimicrobials in the presence of the efflux pump inhibitor, phenylalanine arginine β-naphthylamide (PAβN) (Sigma-Aldrich, St. Louis, Missouri, USA) were determined using a checkerboard assay following the steps outlined in a previous study [47]. Only strains that were resistant to FQs (MIC of ciprofloxacin ≥ 1 mg/L and levofloxacin ≥ 2 mg/L) as well as benzalkonium chloride (MIC ≥ 128 mg/L) and triclosan (MIC ≥ 512 mg/L) were subjected to the checkerboard assay in the presence of efflux pump inhibitors. To address the possibility that a high concentration of PAβN permeabilizes the outer membrane of the cell, a nitrocefin hydrolysis assay was performed as described previously [31,48,49]. In brief, a β-lactamase producing *P. aeruginosa* strain (isolate PA0536 in this study) was treated with PAβN (4 to 128 mg/L) in the presence of nitrocefin (a chromogenic β-lactam) at a final concentration of 32 mg/L. If PAβN causes permeabilization of the outer membrane, nitrocefin will be able to traverse the outer membrane more easily and be exposed to beta-lactamase. This will result in an increased rate of nitrocefin hydrolysis that can be observed as a color change from yellow (≈380 nm) to red (≈490 nm). Polymyxin B, a known outer membrane permeabilizer, was used as a positive control at a final concentration of 128 mg/L. In order to determine the effect of other efflux pumps (not inhibited by PAβN) on the MIC of triclosan, we determined the MIC of triclosan in the presence of carbonyl cyanide-chlorophenylhydrazone (CCCP), which is a proton motive force inhibitor at 12.5 µM using the broth dilution method [50].

### 2.4. Gene Expression Analysis by Reverse-Transcription Quantitative Real-Time Polymerase Chain Reaction (RT-qPCR)

Reverse-transcription quantitative real-time polymerase chain reaction (RT-qPCR) was used to quantify the transcription level of *mexA,* encoding for the periplasmic adapter protein of the MexAB–OprM drug efflux pump. Isolates shown to be resistant to FQ (MIC of ciprofloxacin ≥ 8 mg/L) and BKC (MIC ≥ 128 mg/L) and showing a ≥ 4-fold reduction in the MIC for both antibacterial agents in the presence of PAβN were selected for RT-qPCR analysis. Overnight cultures of *P. aeruginosa* were diluted (1:100) and grown at 37 °C in a cation adjusted Muller Hinton broth, up to the mid-log phase (A_660_ of 0.5) followed by the addition of two times the MIC of ciprofloxacin for 30 min (shock). Total RNA was extracted using a combination of Trizol reagent (Ambion Thermo Fisher Scientific, Carlsbad, CA, USA) and the MN NucleoSpin^®^RNA (Macherey-Nagel GmbH and Co.KG, Duren Germany) kit following the manufacturer’s instructions. Briefly, 1 mL of cells were pelleted and resuspended in Trizol and chloroform, and further purified using the NucleoSpin^®^RNA kit. To remove DNA to a completely undetectable level, RNase-free rDNase digestion and further RNA clean-up was performed using the same kit. RNA purity and concentration were determined using a DeNovix DS-11 + Spectrophotometer (DeNovix Inc., Wilmington, DE, USA) and samples stored at −80 °C. 

RT-qPCR was performed using a magnetic induction cycler instrument (AdeLab Scientific, Adelaide, Australia) with a KAPA SYBR^®^ FAST One-Step RT-qPCR master mix (2×) kit (Sigma-Aldrich, Sigma-Aldrich, St. Louis, Missouri, USA). A housekeeping gene, *rspl*, was used to normalize the transcriptional levels of target genes, which were further calibrated against the *P. aeruginosa* PAO1 control strain. The primer sequences for the target gene (*mexA*) are: *mexA_F* 5′-AGACGGTGACCCTGAATACC-3′; *mexA_R* 5′-GTCGGCCTCGTAGGTGG-3′; *rspl_F* 5′-CCAACGGTTTCGAGGTTTC-3′; *rspl_R* 5′-ACCCTGCTTACGGTCTTTGA-3′. Each PCR experiment was carried out in triplicate with the following cycling parameters; a reverse transcription step at 42 °C for 10 min, and reverse transcription inactivation at 95 °C for 3 min, followed by 40 cycles of PCR at 95 °C for 5 s, 60 °C for 30 s, and 72 °C for 15 s for denaturation, annealing and extension, respectively. A no-template control (NTC) and no reverse-transcriptase control (NRT) were included in each experiment. The relative expression of *mexA* was analyzed using the 2^∆∆cq^ method and normalized against the expression of the PAO1 *rspl* gene [51].

### 2.5. DNA Extraction and Whole Genome Sequencing (WGS)

Genomic DNA was extracted using the MN NucleoSpin^®^Microbial DNA (Machery-Nagel GmbH and Co.KG, Duren, Germany) kit following the manufacturer’s instructions. The quantification of extracted genomic DNA was determined using a DeNovix DS-11 + Spectrophotometer. WGS was conducted at a high-throughput sequencing facility at SA Pathology in South Australia. Sequencing libraries were prepared using the Nextera XT DNA library preparation kit (Illumina Inc., San Diego, Ca, USA), with modifications of the kit’s protocol. Briefly, genomic DNA was fragmented, followed by the amplification of Nextera XT indices (Illumina Inc., San Diego, CA, USA) to the DNA fragments using a low-cycle PCR reaction. The amplicon library was then purified, and normalised manually. Whole-genome sequencing (WGS) was performed on the Illumina NextSeq 550 platform with NextSeq 500/550 Mid-Output kit v2.5 (300 cycles) (Illumina Inc., San Diego, Ca, USA).

### 2.6. WGS Assembly, Annotation and Analysis

Raw paired-end sequencing reads were assembled and annotated using the TORMES pipeline v.1.1 [52]. Multi-Locus Sequence Type (MLST) assignment was carried out using the mlst software (T. Seemann, https://github.com/tseemann/mlst) and the PubMLST database [53]. A pangenome comparison of all isolates was performed using roary2svg (T. Seemann, https://github.com/sanger-pathogens/Roary/blob/master/contrib/roary2svg/roary2svg.pl). Draft genomes were screened for antimicrobial resistance genes using ResFinder [54], Antibiotic Resistance Gene-ANNOTation (ARG-ANNOT) [55], and the comprehensive antibiotic resistance database (CARD) [56]. Genes conferring biocide resistance were identified using the BacMet database (http://bacmet.biomedicine.gu.se) [57]. The *P. aeruginosa* PAO1 genome was used as a reference strain (accession number NC_002516.2; GI 110645304).

Antimicrobial resistance proteins identified by CARD to be carrying a mutation were used in a protein Basic Local Alignment Search Tool (BLASTp) (http://blast.ncbi.nlm.nih.gov/Blast.cgi) to compare regions of similarity between sequences. Protein sequences identified by BLASTp were subsequently used to generate a multi-sequence protein alignment using CLUSTAL O v.1.2.4 [58] to determine the prevalence of the mutation in other proteins.

### 2.7. Genome Accession Numbers

The draft whole genome sequences for 15 *P. aeruginosa* isolates has been deposited at GenBank, the accession number of each isolate were as follows: JACVNM000000000 (PA0115), JACVNN000000000 (PA0404), JACVNO000000000 (PA0461), JACVNP000000000 (PA0471), JACVNQ000000000 (PA0507), JACVNR000000000 (PA0508), JACVNS000000000 (PA0532), JACVNT000000000 (PA0536), JACVNU000000000 (PA0545), JACVNV000000000 (PA0550), JACVNW000000000 (PA0555), JACVNX000000000 (PA0570), JACVNY000000000 (PA0571), JACVNZ000000000 (PA0585), JACVOA000000000 (CLN_26).

### 2.8. Statistical Analysis

Principal component analysis (PCA) was used to cluster isolates based on their antimicrobial resistance patterns, and correlation matrix was used to determine the significant association between biocide and antimicrobial resistance using RStudio, version 3.6.1 [59]. Significant reversion to susceptible MIC in the presence of PAβN was analyzed using the Wilcoxon signed-rank test using IBM SPSS statistical software version 26.0 (SPSS, Armonk, NY, USA). The statistical analysis of *mexA* expression was performed using student t-test and presented in mean ± standard deviation. A *p*-value of ≤ 0.05 was considered significant.

## 3. Results

### 3.1. P. aeruginosa Isolates from Wastewater Display a High Prevalence for Multidrug Resistance in Contrast to Clinical and Veterinary Isolates

**Figure 1:**
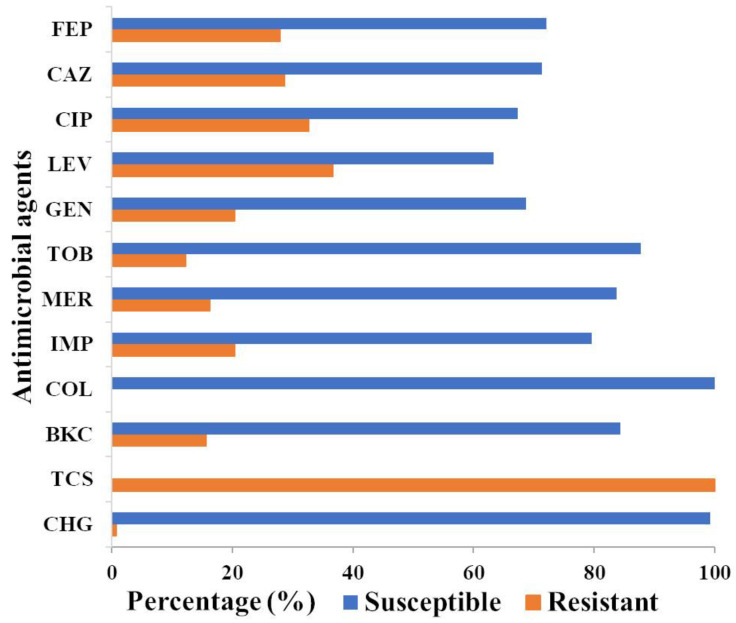
Proportions of antimicrobial susceptibility versus resistance in *P. aeruginosa*. Antimicrobial and biocide susceptibility against 147 *P. aeruginosa* isolates were based on the 2020, European Committee on Antimicrobial Susceptibility Testing (EUCAST) epidemiologic cut-off values (ECOFF) value. The ECOFF values for biocides were as determined in this study (Figure S1), since no ECOFF values were available for biocides against *P. aeruginosa*. The percentage of susceptible and resistant isolates was indicated in orange and blue, respectively. All 147 isolates of *P. aeruginosa* were intrinsically resistant to triclosan. FEP, cefepime; CAZ, ceftazidime; CIP, ciprofloxacin; LEV, levofloxacin; GEN, gentamicin; TOB, tobramycin; MER, meropenem; IMP, imipenem; COL, colistin; BKC, benzalkonium chloride; TCS, triclosan, CHG chlorhexidine digluconate.

A culture collection of 147 *P. aeruginosa* isolates was assembled from clinical samples, veterinary samples, and wastewater, and their susceptibility against five different classes of antimicrobials was determined. The highest frequency of resistance was shown to levofloxacin (36.7%), followed by ciprofloxacin (32.7%), ceftazidime (28.6%), cefepime (27.9%), imipenem (20.4%), gentamicin (20.4%), meropenem (16.3%), and tobramycin (12.2%) (Figure 1 and Table S1). None of the isolates were resistant to colistin. Twenty-eight (19%) isolates were resistant to at least three classes of antimicrobials and were defined as MDR. Of these, 53.6% originated from wastewater.

### 3.2. P. aeruginosa Has a High Resistance against Common Biocides Used in Clinical Settings

Since antimicrobial resistance is often associated with an increased resistance to biocides, the resistance of *P. aeruginosa* against common biocides of clinical importance was investigated. There are currently no ECOFF values for biocides against *P. aeruginosa*; hence, the susceptibility of the 147 *P. aeruginosa* isolates against the three biocides, BKC, triclosan, and chlorhexidine digluconate, was established to determine the MIC distribution and to establish preliminary ECOFF values as a reference point for measuring resistance (non-wild type). Interestingly, almost all *P. aeruginosa* isolates were intrinsically resistant to triclosan with an MIC of > 1024 mg/L, only three clinical isolates showed a MIC value ranging from 64 to 256 mg/L (Figure 1, Table S1). The MIC data for chlorhexidine digluconate and BKC exhibit normal distributions, ranging between three and five dilution steps and justified the criteria set by EUCAST for establishing ECOFF values with ECOFF values of 32 and 128 mg/L, respectively (Figure S2). Only one clinical strain displayed resistance to chlorhexidine digluconate, while twenty-three (15.6%) of the isolates had shown an MIC above the wild-type ECOFF value of 128 mg/L to BKC and hence were deemed BKC resistant.

### 3.3. Phenotypic Correlation between Biocide and Antimicrobial Resistance in P. aeruginosa Isolates

**Figure 2:**
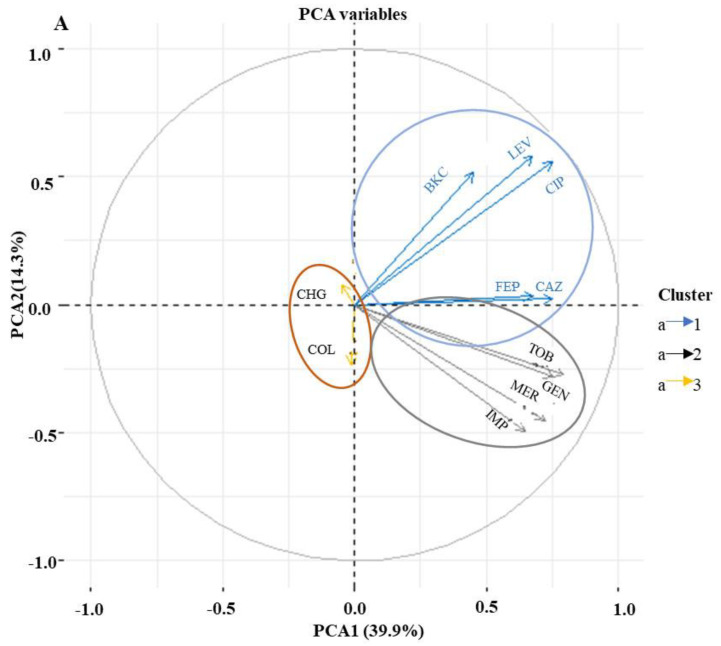

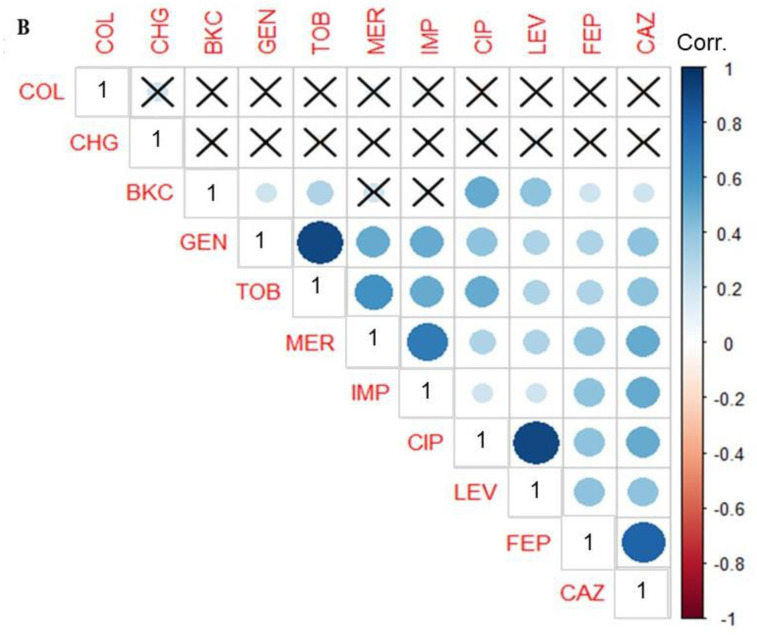
BKC resistance correlate with antimicrobial resistance. Principal component analysis (PCA) and Pearson correlation coefficient of 147 *P. aeruginosa* isolates (variables) against five classes of antimicrobials and two biocides based on minimum inhibitory concentration (MIC) value (scores). Colors are used to differentiate isolates with similar clusters of antimicrobial susceptibility patterns (**A**). The correlation matrix indicates a significant correlation between BKC and antimicrobial classes FQs, cephalosporins, and aminoglycosides. The intensity of the blue circles indicates the correlation coefficient value, and the sizes indicate the significance of correlation (*p* < 0.05). The bar to the right of the figure indicates the color coding of the correlation value from +1 (strong positive correlation) to −1 (strong negative correlation). Coordinates marked with “×” indicate no significant correlation (*p* value > 0.05) (**B**). CAZ, ceftazidime; FEP, cefepime; LEV, levofloxacin; CIP, ciprofloxacin; IMP, imipenem; MER, meropenem; TOB, tobramycin; GEN, gentamicin; COL, colistin; BKC, benzalkonium chloride; CHG chlorhexidine.

In order to establish if any relationship exists between antimicrobial and biocide resistance, a PCA was performed. Analyses of the MIC values using PCA revealed three clusters of antimicrobial resistance in *P. aeruginosa* isolates (Figure 2A). The first cluster showed isolates’ resistance to BKC, FQs, and cephalosporins. The second cluster includes isolates resistant to aminoglycosides and carbapenems, while the third cluster exhibits isolates susceptible to colistin and chlorohexidine digluconate (Figure 2A). The MIC values of triclosan were not included in the PCA analysis due to the intrinsic resistance of *P. aeruginosa* against triclosan (Table S1). To further investigate the dependence between multiple variables at the same time, a correlation matrix was used. The result showed a significant correlation between BKC resistance and the antimicrobial classes FQs, cephalosporins and aminoglycosides (Figure 2B). Since a large number of BKC-tolerant isolates were also resistant to FQs as shown in Figure 2A, further investigation into the underlying mechanisms for cross-resistance between BKC tolerant and ciprofloxacin-resistant subpopulations of *P. aeruginosa* isolates was carried out.

### 3.4. Efflux Pump Inhibition Reveals RND Pump Mediated Biocide and Antimicrobial Resistance

**Figure 3:**
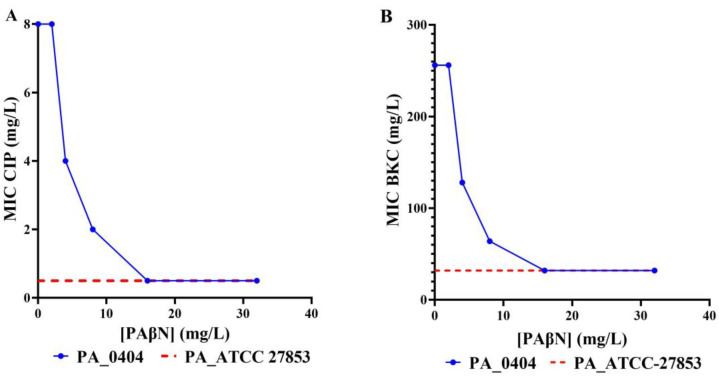
Reversal of resistance in the presence of an efflux pump inhibitor. Effect of efflux pump inhibitor, (PaβN) in the CIP-resistant and BKC-resistant PA0404 isolate in comparison to a susceptible strain. The MIC values for CIP (**A**) and BKC (**B**) of the CIP and BKC-resistant PA0404 isolate were determined in the presence of increasing concentrations of the efflux pump inhibitor PAβN (blue line). For reference, the MIC for the wild-type reference strain—*P. aeruginosa* ATCC—27853 are indicated, too (red broken line).

In order to investigate the putative role of drug efflux pumps in the observed antimicrobial and biocide resistance, the effect of the efflux pump inhibitor PAβN on BKC, triclosan, and ciprofloxacin resistance was determined. All BKC-tolerant and 37.5% of the ciprofloxacin-resistant *P. aeruginosa* isolates were reversed back to sensitive levels when tested in the presence of PAβN (example Figure 3, Table S2). However, PAβN did not modify the MIC of triclosan for any of the isolates tested (Table S2). To explore other potential efflux pumps that may be responsible for triclosan intrinsic resistance but not inhibited by PAβN, we used CCCP. Nonetheless, the reversion of triclosan resistance was not shown upon the addition of CCCP, either (Table S2). To verify that the observed reduction in the MIC of BCK and ciprofloxacin in the presence of PAβN at 32 mg/L was as a direct result of inhibition of the RND-type drug efflux pumps and not due to any non-specific outer membrane permeability effects, the integrity of the outer membrane was determined in the presence of PAβN at 32 mg/L using the nitrocefin hydrolysis rate as an indicator of outer membrane integrity [47,49]. The results clearly indicate no increase in the nitrocefin hydrolysis rate in the presence of PAβN; hence, the reduction in MIC values observed with PAβN was solely due to the inhibition of active efflux (Figure S2).

### 3.5. RT-qPCR Revealed MexAB-OprM efflux Pump Overexpression

**Figure 4:**
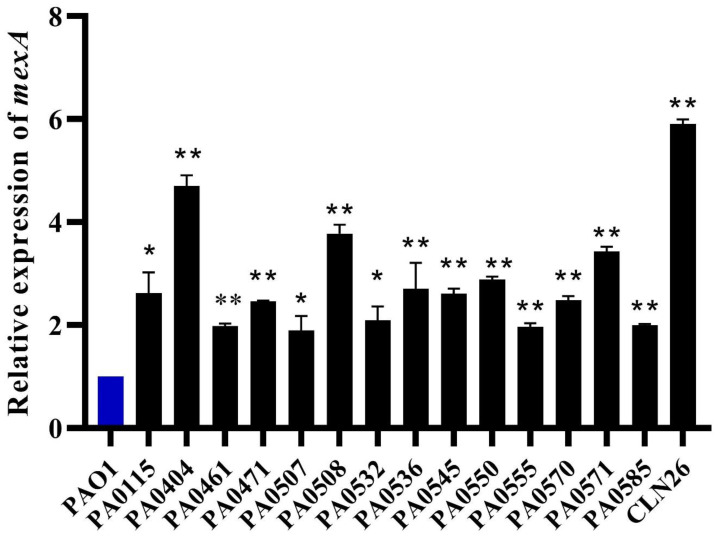
*MexA* is overexpressed in BKC-tolerant and ciprofloxacin-resistant isolates. The expression levels of the *mexAB-oprM* operon, as assessed by RT-qPCR of the *mexA* gene, reveal the overexpression of *mexAB-oprM* in BKC-tolerant and CIP-resistant isolates (black bars) as compared to the wild-type PAO1 strain (blue bar) (set at a value of 1, by definition). The results are presented in mean ± standard deviation. Statistical analysis was performed using Student t-test and statistical significance was represented with asterisks (*) as shown in the figure (* *p* < 0.01; ** *p* < 0.001).

Due to the effect of the efflux pump inhibitor PAβN on ciprofloxacin and BKC resistance, it was hypothesized that the cross-resistance between these compounds could be mediated by the overexpression of multidrug efflux pumps. MexAB-OprM is the most promiscuous and constitutively expressed efflux pump that has a significant role in natural resistance of *P. aeruginosa*. To verify the role of MexAB-OprM in cross-resistance, 15 isolates showing an MIC reduction by at least 4-fold in the presence of PAβN were selected for RT-qPCR. Results revealed an overexpression of the MexA pump, showing a 1.896- to 5.911-fold (mean 3.1) difference between the 15 isolates examined and the wild-type PAO1 strain (Table 1, Figure 4). Almost all MexAB-OprM overexpressing isolates are resistant to BKC and showed cross-resistance to FQs resistance, cephalosporins resistance, and MDR subpopulations. The highest level of overexpression (≈6 fold) of MexA was observed for the clinical isolate. Since this clinical isolate was isolated from cystic fibrosis patients, the high-level overexpression is consistent with the role of MexAB-OprM in invasion [60] in addition to its role in antimicrobial resistance.

### 3.6. WGS Analysis Reveals Multiple Mutations in the Regulators of the mexab-oprM Efflux Pump

**Figure 5:**
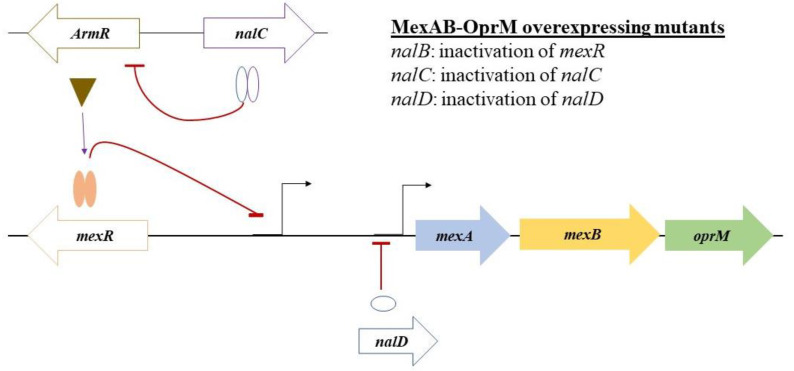
Schematic representation of the regulator genes involved in the repression of MexAB-OprM efflux pump in *P. aeruginosa*. The basal expression of the MexAB-OprM efflux pump in wild-type strains is controlled by a local repressor *mexR* (self-regulated) by binding as a homodimer to the distal promoter of the *mexAB-oprM*. A second repressor, NalD, directly binds as a monomer to the proximal promoter of the *mexAB-oprM* Operon. The third repressor, *nalC*, has an indirect effect on *mexAB-oprM* expression; it represses the expression of gene *armR*, the product of which acts as an anti-repressor of *mexR*. Mutations in these repressors result in a high-level expression of MexAB-OprM, rendering stable, acquired resistance. Mutations in these three repressors have been reported in *mexR (nalB mutation), nalD*, and *nalC* mutants, respectively (Table 1).

WGS analysis was carried out to confirm the molecular mechanism for the link between biocide and FQ resistance in the same 15 *P. aeruginosa* isolates as mentioned above. Antimicrobial resistance genes were identified using three databases with the CARD identifying not only AMR genes and proteins but also AMR protein variants and their mutations [56]. Further interrogation of the WGS data with blast analysis and multiple sequence alignment revealed multiple amino acid mutations in the regulatory genes of the *mexAB-oprM* efflux pump (Table 1, Figure 5), which may lead to the observed increased expression of this efflux pump (Figure 4) and subsequent high-level resistance to BKC and FQs. Of these, three non-synonymous mutations were found in NalC proteins, including G71E, S209R, and E153Q, and one mutation (V126E) was found in the MexR regulator. In addition to these mutations, isolate PA0570 had a 13 amino acid truncation at the beginning of the NalC regulator protein. Isolate PA0404, with a fairly high overexpression fold of 4.7, contained an additional T11N mutation in the NalD repressor protein and displayed a novel combination of mutations in the MexR, NalC, and NalD proteins (Table 1).

WGS analysis also revealed that two isolates, PA0508 and PA0532, with MIC values for BKC equal to the ECOFF value, lack the wild-type *mexEF-oprJ* MDR efflux system and its corresponding regulator genes, *mexT* and *mexS*. None of the isolates displayed any mutations in the amino acid sequence of the regulator genes of the *mexCD-oprN, mexEF-oprJ*, or *mexXY-oprM* efflux pump systems.

Collectively, the data confirm MexAB-OprM efflux pump overexpression and its role in the cross-resistance of BKC and FQ.

### 3.7. The Role of Other Resistance Gene Determinants in the Development of High-Level Resistance to FQs and Biocides

Given that 14 sequenced *P. aeruginosa* isolates displayed high levels of FQ resistance that could not be completely reversed by the efflux pump inhibitor PAβN (Table S2), we investigated the role of other resistance mechanisms using WGS. All 15 of these *P. aeruginosa* isolates harbored mutations in the DNA gyrase and/or topoisomerase IV proteins (GyrA/B and ParC/E). The main amino acid mutations noted were T83I and D87N in GyrA, S87L and V297I in ParC, and D533E in ParE. A 33 amino acid truncation of the ParC protein was also observed in one isolate (PA0571) as well as a one amino acid deletion in the ParE protein (PA0570). One isolate (CLN26) carried two mutations in the GyrB protein (E468D and H148N) and another carried two mutations in the ParE protein (P438S and L501F) in combination with a T83I mutation in GyrA (Table 1). Although showing no difference to the level of resistance, the ciprofloxacin-modifying *crpP* gene was also identified in two of the isolates (PA0536 and PA0545). A single amino acid mutation in the GyrA/B or ParC/E (conserved mutations, as observed in these and other *P. aeruginosa* isolates) is seldom sufficient to confer high-level FQ resistance. However, it is possible that the cumulative effect of these mutations could contribute to the high level of FQ resistance observed.

To screen the presence of resistance genes to BKC and triclosan, the BacMet database was used. All isolates that are resistant to triclosan were shown to carry the triclosan-resistant enoyl-acyl-carrier protein reductase *fabV* gene encoding for the FabV protein (Table 1).

### 3.8. The Pangenome Analysis Indicated an Evolutionary Divergence between mexAB-oprM Efflux Pump Overexpressing P. aeruginosa Isolates

To decipher the genetic relationships among MDR *P. aeruginosa* isolates overexpressing the *mexAB-oprM* efflux pump system, whole-genome MLST and pangenome analysis was conducted on 15 *P. aeruginosa* isolates. Three different MLSTs were identified in the study, twelve isolates of which were represented by a single ST, which is the known epidemiological high-risk clone ST235. All of the isolates belonging to ST235 were recovered from wastewater. The remaining *P. aeruginosa* isolates belonged to ST815 (*n* = 2), also isolated from wastewater and ST274 (*n* = 1) isolated from a clinical sample. To further confirm that the dominant ST235 isolates are not diffusion of the same clone as a result of antimicrobial selection pressure, a pangenome analysis was carried out, with the results showing an evolutionary divergence between ST235 isolates (Figure S3).

## 4. Discussion

Our study on a collection of *P. aeruginosa* isolated from diverse ecological niches revealed a relatively high proportion (19%) of MDR isolates. This number is much higher than what the Australian Group on Antimicrobial Resistance surveillance found in bacteremic patients (4.3%) [61] and higher than the European Union and European Economic Area surveillance population-weighted mean resistance (12.8%) [62]. The reason for the high proportion of MDR could be the inclusion of isolates from healthcare-generated wastewater which accounted for more than half of the MDR isolates in our study. Multiple factors could contribute to the increased antimicrobial resistance observed in isolates from healthcare wastewater, such as high antimicrobial and disinfectant use in healthcare settings, as well as patients on prolonged courses of antimicrobials who could harbor and shed resistant isolates [19,63].

Most of our isolates originating from wastewater environments demonstrated increased resistance to BKC and cross-resistance for FQ and MDR (55.5% of the total wastewater isolates). This fraction is significantly higher than that observed for isolates from other ecological niches. This difference could be attributed to the difference in the concentration and length of exposure to these agents in these two environments. In the clinical environment, biocides and antibiotics are used at inhibitory concentrations and are less likely to select tolerant clones [64], whereas residues exist at subinhibitory concentrations in the wastewater environment that could provide a selective pressure for the development of resistant subpopulations to thrive and predominate [65].

Studies on laboratory-adapted biocide insensitive *P. aeruginosa* indicated a correlation with antimicrobial resistance with efflux pump overexpression as the main mechanism of resistance [17,24,66,67,68] and reviewed in [43,69]. These observations have led to the hypothesis that there could be a correlation between biocide use and the development of antimicrobial resistance in clinical or environmental situations, although this has never been translated from laboratory-adapted strains to a confirmation in the natural environment. Our study is the first to provide evidence for the correlation between biocide and antibiotic cross-resistance driven by efflux pump overexpression on isolates from native niches that have “adapted” to their environment. It is also the first study of naturally developed cross-resistance that confirms findings previously only seen in laboratory-based studies. Our work also bestows credence to previous suggestions that healthcare-associated effluent could act as a hotspot for antimicrobial resistance development [39]. The reversal of resistance by the efflux pump inhibitor PAβN, direct measurement of efflux pump expression, and WGS confirmed mutational hyperexpression of the chromosomally encoded MexAB-OprM efflux pump as the main reason for the observed resistance and cross-resistance to antibiotics. The upregulation of MDR efflux pumps provides a fitness advantage that is central to the survival of an organism at low concentrations of a diverse range of xenobiotics such as is found in the wastewater environment [17,24,26,70].

Whole-genome analysis of MLSTs revealed an overrepresentation of a subpopulation of *P. aeruginosa* wastewater isolates belonging to ST235, which has been described as an MDR gene-enriched international high-risk clone isolated from wastewater [3], human, animal, and other niches [8]. Studies suggest that the global spread of ST235 has been potentially associated with the selective pressure of FQs [9]. Interestingly, we also identified two wastewater isolates that belong to ST815, which appear to be uncommon but are resistant to four major antipseudomonal drugs, including carbapenems. The only record of ST815 comes from a study in Portugal [71]. These isolates also carried the ciprofloxacin-modifying *crpP* resistance gene (50, 51), which has been shown to marginally increase MICs to FQs through ciprofloxacin phosphorylation [72]. The reduced susceptibility mediated by this enzyme is not to the level of clinical resistance; however, the presence of this enzyme facilitates the selection of mutants that generate quinolone resistance and promote treatment failure [36]. Only a single clinical isolate identified from a cystic fibrosis patient showing cross-resistance between BKC and FQs belongs to ST274. This ST is another epidemic high-risk clone circulating in European countries, mainly Spain, and Australia and has previously been isolated from both cystic and non-cystic fibrosis patients [73,74].

Finally, we found that every *P. aeruginosa* isolate in this study exhibits an intrinsic resistance to triclosan. Sequencing confirmed the presence of the *fabV* gene, coding for the triclosan-resistant enoyl-acyl-carrier protein in each of these isolates [42]. Before the US Food and Drug Administration banned the use of triclosan (www.fda.gov) in 2016, triclosan was extensively used in common household products such as antimicrobial soaps and other cleaning products. Given the propensity of triclosan to select for AMR [70], this move would help prevent the further development of triclosan and concomitant AMR. However, due to the acquisition of the *fabV* gene conferring high-level and stable triclosan resistance, the proportion of triclosan-resistant populations appears to be stable [42]. Biocides are also frequently used in mass-marketed household products, which could lead to the development of resistance and have severe consequences for their clinical usefulness [75,76]

Our study clearly shows that cross-resistance between BKC and FQ, as well as MDR, are driven by hyperexpression of the drug efflux pumps and occurs spontaneously in a natural environment. This should serve as a warning that the unlimited and uncontrolled use of biocides has repercussions as BKC plays a role in the development of resistance against our most useful classes of antimicrobials used to treat resistant Gram-negative infections. It also shows the power of efflux pumps as mechanisms of resistance that continue to evolve, ever expanding their capacity to protect bacterial cells against the very antimicrobials we use for treatment. As such, greater restrain in the use of biocides is needed to preserve the usefulness of these important chemicals and to extend the lifetime of currently used antimicrobials.

## Figures and Tables

**Table 1:** Phenotypic and genotypic characteristics of the 15 MexAB-OprM efflux pump overexpressing *P. aeruginosa* isolates, determined by using conventional MICs method, RT-qPCR, and WGS.

Strains	ST	Antimicrobial Susceptibility									RP	MexAB-OprM	Fluoroquinolone Resistance Determinants					Mutation in MexAB-OprM Efflux Pump Regulators			Triclosan
		FEP	CAZ	CIP	LEV	GEN	TOB	MER	IMP	COL			GyrA	GyrB	ParC	ParE	CrpP	MexR	NalC	NalD	FabV
PAO1		S	S	S	S	S	S	S	S	S	S	1									
PA0115	235	R	R	R	R	R	S	S	S	S	MDR	2.622	T83I	−	S87L	D533E	−	V126E	S209R, G71E, E153Q	−	P36L
PA0404	235	R	R	R	R	R	R	S	R	S	MDR	4.705	T83I	−	S87L	D533E	−	V126E	S209R, G71E, E153Q	T11N	P36L
PA0461	235	R	R	R	R	R	R	R	R	S	MDR	1.986	T83I	−	S87L	D533E	−	V126E	S209R, G71E, E153Q	−	P36L
PA0471	235	R	R	R	R	S	S	R	R	S	MDR	2.462	T83I	−	S87L	D533E	−	V126E	S209R, G71E, E153Q	−	P36L
PA0507	235	R	R	R	R	R	S	S	S	S	MDR	1.896	T83I	−	S87L	D533E	−	V126E	S209R, G71E, E153Q	−	P36L
PA0508	235	R	R	R	R	R	R	S	S	S	MDR	3.778	T83I	−	S87L	D533E	−	V126E	S209R, G71E, E153Q	−	P36L
PA0532	235	R	R	R	R	R	R	R	R	S	MDR	2.097	T83I	−	S87L	D533E	−	V126E	S209R, G71E	−	P36L
PA0536	815	R	R	R	R	R	R	R	R	S	MDR	2.707	D87N	−	V297I	D533E	+	V126E	S209R, G71E	−	P36L
PA0545	815	R	R	R	R	R	R	R	R	S	MDR	2.610	D87N	−	V297I	D533E	+	V126E	S209R, G71E	−	P36L
PA0550	235	R	R	R	R	R	S	S	S	S	MDR	2.882	T83I	−	S87L	D533E	−	V126E	S209R, G71E, E153Q	−	P36L
PA0555	235	R	R	R	R	S	S	S	S	S	NMDR	1.969	T83I	−	S87L	D533E	−	V126E	S209R, G71E, E153Q	−	P36L
PA0570	235	R	R	R	R	R	S	S	S	S	MDR	2.486	T83I	−	S87L, TR*	D533E	−	V126E	S209R, G71E, E153Q, TR***	−	P36L
PA0571	235	R	R	R	R	R	S	S	S	S	MDR	3.427	T83I	−	S87L	D533EE569-**	−	V126E	S209R, G71E, E153Q	−	P36L
PA0585	235	R	R	R	R	R	S	S	S	S	MDR	1.996	T83I	−	S87L	D533E	−	V126E	S209R, G71E, E153Q	−	P36L
CLN_26	274	R	R	R	R	S	S	S	S	S	NMDR	5.911	T83I	E468D, H148N	−	P438S, L501F	−	−	S209R, G71E	−	P260T

CLN: clinical strains; PA: wastewater strains; PAO1: reference strain; ST, sequence type; RP: resistance profile; S: susceptible; NMDR: non-multidrug resistant; MDR: multidrug resistant; E569-** one amino acid deletion at 569 amino acid position of ParE; TR*: 33 amino acid truncation at the C-terminal; TR*** 13 amino acid truncation at the N-terminal;−: no mutation; V: valine; P: proline; T: threonine; D: aspartate; L: leucine; S: serine; E: glutamate; Q: glutamine; I: isoleucine; R: arginine G: glycine; F: phenylalanine; WGS: whole genome sequencing; MIC: minimum inhibitory concentration; RT-qPCR: reverse-transcription quantitative real-time polymerase chain reaction; CAZ: ceftazidime; CIP: ciprofloxacin; COL: colistin; GEN: gentamicin; FEP: cefepime; IMP: imipenem; LEV: levofloxacin; MER: meropenem; TOB: tobramycin.

## Supplementary Materials

The following are available online at https://www.mdpi.com/2076-2607/8/11/1647/s1, **Table S1**. Minimum inhibitory concentration distribution of antimicrobial agents against *P. aeruginosa* (*n* = 147) isolates collected from clinical, veterinary and wastewater, **Table S2.** Effects of PAβN on the MIC value of P. aeruginosa isolates resistant to an antibiotic (e.g., ciprofloxacin) and biocides: BKC and triclosan. **Figure S1**: ECOFF value determinations for chlorohexidine digluconate and BKC, **Figure S2**. Evaluating the outer membrane-permeabilizing activity of PAβN, **Figure S3**. Visualization of pangenome-analysis of 15 *P. aeruginosa* isolates.
