# Supplementary material for: Efflux Pump-Driven Antibiotic and Biocide Cross-Resistance in Pseudomonas aeruginosa Isolated from Different Ecological Niches: A Case Study in the Development of Multidrug Resistance in Environmental Hotspots
Source: Microorganisms. 2020 Oct 24;8(11):1647. doi: 10.3390/microorganisms8111647 (PMC7690850; doi:10.3390/microorganisms8111647)
Supplement: Supplementary file 1 [file microorganisms-08-01647-s001.pdf]

Supplementary Material

Minimum inhibitory concentrations (MICs) of *P. aeruginosa* against five antibiotic classes (9 antibiotics) and three biocides are depicted in **Table S1**.

Phenotypic efflux pump activity was determined in the presence of efflux pump inhibitors (EPIs), phenylalanine arginine β-naphthylamide (PAβN) or carbonylcyanide *m*-chlorophenylhydrazone (CCCP) against *P. aeruginosa* isolates. No reduction in MIC of triclosan to either of the EPIs and significant reduction in MIC of BKC and CIP in the presence of PAβN compared with the absence are shown in **Table S2.** Additionally, the percentage of isolates that reversed back to the values observed for the sensitive strain to the corresponding antimicrobial agent in the presence of PAβN was shown. Laboratory strain wild type PAO1 (negative control) was used. CLN: clinical isolates; PA: wastewater isolates; Dog: Veterinary (dog otitis media) isolates.

Table S1. MIC distribution of antimicrobial agents against *P. aeruginosa* (*n* = 147) isolates collected from clinical, veterinary and wastewater samples.

**Antimicrobial Agent**	**Percent of *P. aeruginosa* Isolates with MIC (mg/L) at:**														
	**0.06**	**0.125**	**0.25**	**0.5**	**1**	**2**	**4**	**8**	**16**	**32**	**64**	**128**	**256**	512	1024
**Cefepime**					4.1	13.6	23.1	31.3	12.2	8.2	5.4	2.1			
**Ceftazidime**				1.4	1.4	32	24.5	12	5.4	6.1	11.6	4.1	1.4		
**Ciprofloxacin**	11.6	19.0	22.4	14.3	6.1	4.1	4.8	4.8	2.0	10.9					
**Levofloxacin**		1.4	8.2	17.7	36.1	3.4	9.5	4.1	6.1	8.8	4.8				
**Gentamycin**			0.7	0.7	14.3	23.8	29.9	15.6	2.7	4.8	0.7	2.0	4.8		
**Tobramycin**		5.4	15.6	34	19.7	12.9	2	2.7	0.7	1.4	2	3.4			
**Meropenem**	3.4	4.8	17.7	21.1	22.4	14.3	6.8	3.4	2.7	2	0	1.4			
**Imipenem**			1.4	2	11.6	31.3	33.3	7.5	4.8	3.4	2.7	2.1			
**Colistin**		0.7	4.1	40.8	40.1	13.6	0.7								
**Chlorhexidine digluconate**							5.4	25	52.4	16.3	0.7				
**Triclosan**											0.7	0.7	0.7		97.9
**Benzalkonium chloride**										3.4	73.5	7.5	15.0	0.7	

Vertical black lines indicate EUCAST ECOFF values and red lines indicate ECOFF values for biocides as determined in this study (Figure S1). MICs of isolates greater than the highest concentration of antimicrobials tested are presented in the next highest dilution in the shaded region and reported as greater than or equal to that MIC. Triclosan ECOFF values were difficult to establish because the MIC value of 97.9% is >1024mg/L.

Table S2. MICs in the presence and absence of efflux pump inhibitors showing significant reduction to ciprofloxacin and BKC in the presence of PAβN but not to triclosan.

		MIC								
**Stains**	**CIP (n=48)**	**CIP+ PAβN**	**FR**	**BKC (n=23)**	**BKC+ PAβN**	**FR**	**TCS (n=53)**	**TCS+ PAβN**	**FR**	**TCS+ CCCP**
PA0115	32	4	8	256	32	8	> 512	> 512	1	>512
PA0397	0.25	ND		256	128	2	> 512	> 512	1	>512
PA0398	0.25	ND		512	128	4	> 512	> 512	1	>512
PA0404	8	0.5	16	256	32	8	> 512	> 512	1	>512
PA0449	1	0.5	2	64	ND		> 512	> 512	1	>512
PA0461	32	4	8	256	32	8	> 512	> 512	1	>512
PA0471	32	4	8	256	32	8	> 512	> 512	1	>512
PA0507	32	4	8	256	32	8	> 512	> 512	1	>512
PA0508	32	2	16	128	32	4	> 512	> 512	1	>512
PA0532	32	2	16	128	32	4	> 512	> 512	1	>512
PA0536	32	2	16	256	32	8	> 512	> 512	1	>512
PA0540	1	0.25	4	256	64	4	> 512	> 512	1	>512
PA0541	2	0.5	4	256	64	4	> 512	> 512	1	>512
PA0542	0.25	ND		256	32	8	> 512	> 512	1	>512
PA0544	8	0.25	32	256	64	4	> 512	> 512	1	>512
PA0545	32	4	8	256	32	8	> 512	> 512	1	>512
PA0546	32	4	8	256	32	8	> 512	> 512	1	>512
PA0548	0.06	ND		256	128	2	> 512	> 512	1	>512
PA0550	32	8	4	256	32	8	> 512	> 512	1	>512
PA0555	32	4	8	256	32	8	> 512	> 512	1	>512
PA0570	32	8	4	256	64	4	> 512	> 512	1	>512
PA0571	32	8	4	256	64	4	> 512	> 512	1	>512
PA0585	32	4	8	256	64	4	> 512	> 512	1	>512
CLN2	4	1	4	64	ND		> 512	> 512	1	>512
CLN 7	0.125	ND		128	32	4	> 512	> 512	1	>512
CLN 13	8	8	1	64	ND		> 512	> 512	1	>512
CLN 14	32	32	1	64	ND		> 512	> 512	1	>512
CLN 19	32	16	2	128	ND		> 512	> 512	1	>512
CLN 24	2	0.06	32	64	ND		> 512	> 512	1	>512
CLN 25	2	0.125	16	128	ND		> 512	> 512	1	>512
CLN 26	16	4	4	256	32	8	> 512	> 512	1	>512
CLN 28	16	4	4	64	ND		> 512	> 512	1	>512
CLN 29	4	0.5	8	64	ND		> 512	> 512	1	>512
CLN 30	8	0.5	16	64	ND		> 512	> 512	1	>512
CLN 32	4	0.5	8	64	ND		> 512	> 512	1	>512
CLN 33	4	0.5	8	64	ND		> 512	> 512	1	>512
CLN 34	8	8	1	32	ND		> 512	> 512	1	>512
CLN 36	8	8	1	64	ND		> 512	> 512	1	>512
CLN 38	8	1	8	64	ND		> 512	> 512	1	>512
CLN 41	4	0.5	8	64	ND		> 512	> 512	1	>512
CLN 62	8	8	1	64	ND		> 512	> 512	1	>512
CLN 64	8	2	4	64	ND		> 512	> 512	1	>512
CLN 65	1	1	1	64	ND		> 512	> 512	1	>512
CLN 66	8	1	8	64	ND		> 512	> 512	1	>512
CLN 71	8	1	8	64	ND		> 512	> 512	1	>512
CLN 72	8	1	8	64	ND		> 512	> 512	1	>512
CLN 76	0.06	ND		256	32	8	> 512	> 512	1	>512
CLN 81	4	0.5	8	64	ND		> 512	> 512	1	>512
CLN 84	8	0.5	16	64	ND		> 512	> 512	1	>512
CLN 86	16	0.5	32	64	ND		> 512	> 512	1	>512
Dog 3	2	2	1	64	ND		> 512	> 512	1	>512
Dog 7	2	0.25	8	64	ND		> 512	> 512	1	>512
Dog15	1	0.125	8	64	ND		> 512	> 512	1	>512
Dog23	1	0.25	4	64	ND		> 512	> 512	1	>512
**Reversed to sesuptible**		18 (37.5)			23 (100)			0		
**Laboratory strains**										
PA-ATCC 27853	0.5	0.5	1	32	32	1				

CIP: ciprofloxacin; BKC: benzalkonium chloride; TCS: triclosan; PAβN: phenylalanine arginine b-naphthylamide; FR: fold reduction; ND: not done, PA-ATCC27853: *P. aeruginosa* susceptible

control strain; **CCCP**: carbonylcyanide *m*-chlorophenylhydrazone to verify the presence of other efflux pump.

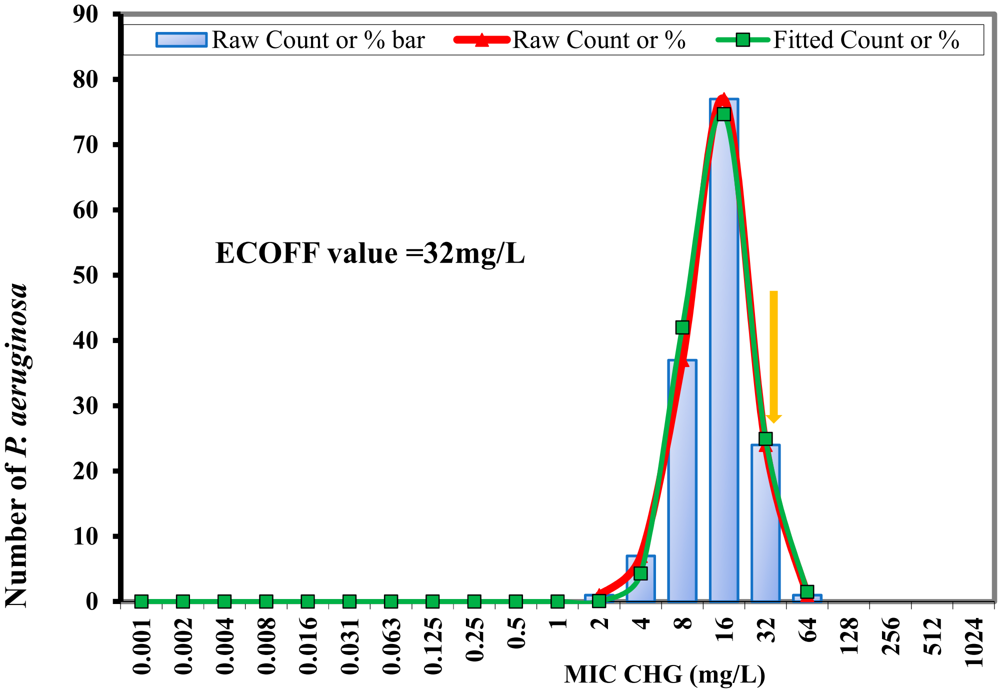

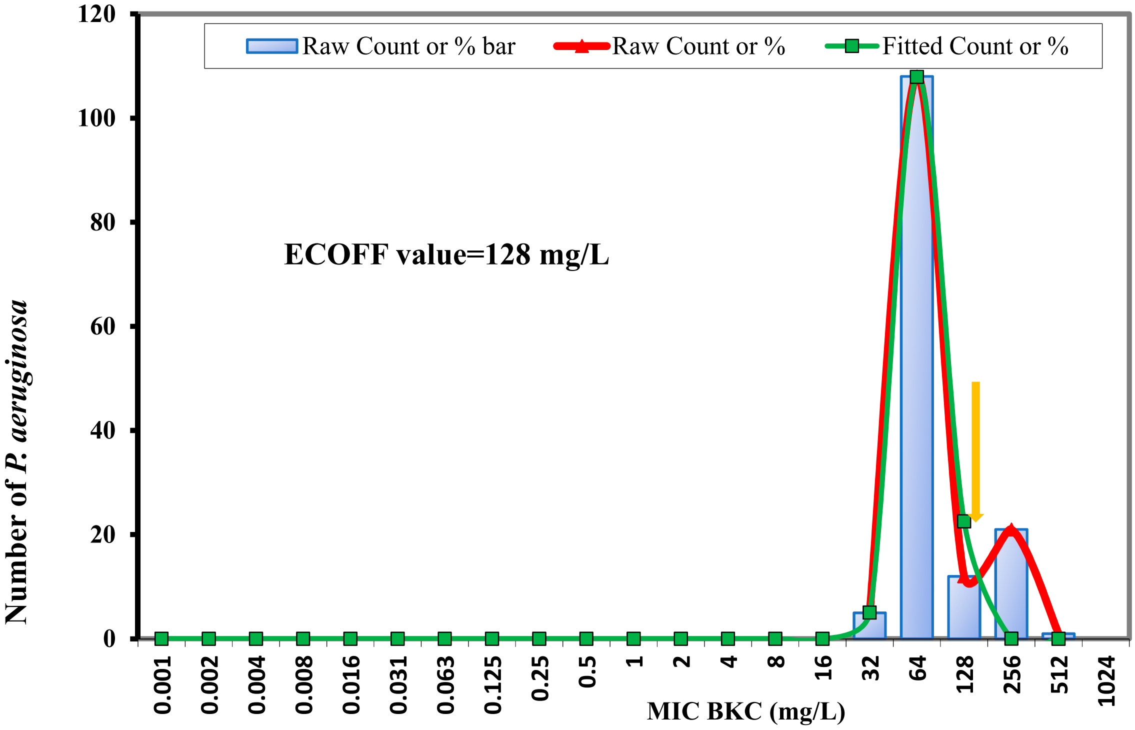

Figure S1. ECOFF value determination for CHG and BKC. ECOFF values for the *P. aeruginosa*-biocide combination according to EUCAST, 2019. The yellow arrow indicates the point of ECOFF value. BKC, Benzalkonium chloride; CHG, Chlorhexidine digluconate

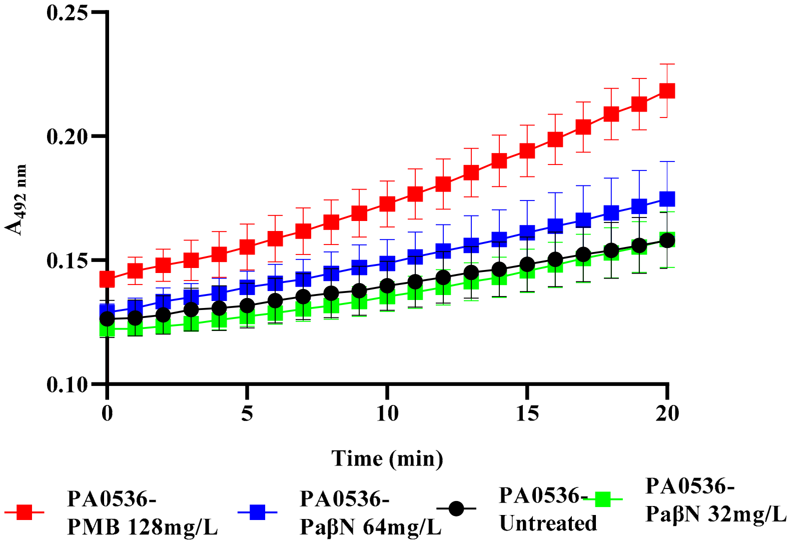

Figure S2. PAβN at 32 mg/L does not permeabilize the outer membrane. The outer membrane-permeabilizing activity of PAβN was measured as an increase in initial rates of nitrocefin hydrolysis by β-lactamase producing *P. aeruginosa* (PA0536) isolates in this study. Black dots represent untreated cells whilst the green, blue and red squares represent treatment with PAβN at 32 mg/L, 64 mg/L and polymyxin B at 128mg/L, respectively. The absorbance of nitrocefin hydrolysis rate was monitored over time by monitoring the increase in absorbance at 492 nm. The assay was performed in triplicate and results are presented with mean± standard error of the mean (SEM).

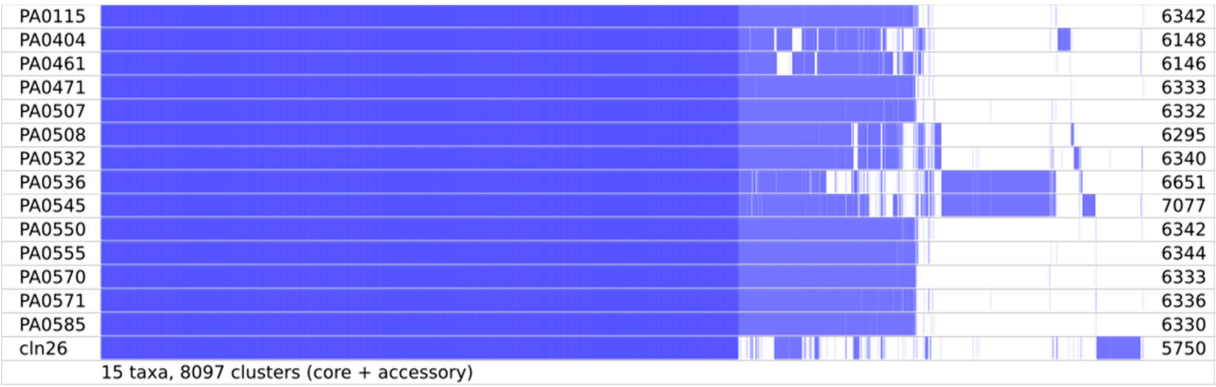

Figure S3. Visualization of pangenome-analysis of 15 *P. aeruginosa* isolates. A schematic presentation showing presence (color) or absence (blank) of genes between isolates. Deep blue

color to the left (highly conserved core genome) and light blue to the right (accessary genome). Pangenome analysis revealed the evolutionary divergence of all 15 *P. aeruginosa* isolates.
